# Performance Evaluation of Warm Asphalt Mixtures Containing Chemical Additive and Effect of Incorporating High Reclaimed Asphalt Content

**DOI:** 10.3390/ma14143793

**Published:** 2021-07-07

**Authors:** Mukul Rathore, Viktors Haritonovs, Martins Zaumanis

**Affiliations:** 1 Celu Eksperts, LV-5052 Ikskile, Latvia; 2 Department of Roads and Bridges, Riga Technical University, LV-1048 Riga, Latvia; viktors.haritonovs@rtu.lv; 3 EMPA Swiss Federal Laboratories for Materials Science and Technology, CH-8600 Dubendorf, Switzerland; martins.zaumanis@empa.ch

**Keywords:** WMA, RA, RAP, chemical additive, short term aging, asphalt mixture, TSRST, rutting, cracking, moisture susceptibility

## Abstract

Reclaimed asphalt (RA) and Warm mix asphalt (WMA) are two widely used environmentally friendly mixtures in the paving industry. This study compares the laboratory performance of conventional hot mix asphalt (HMA) with virgin WMA, and WMA containing 60% RA content, using thermal stress restrained specimen test, wheel tracking test, and indirect tensile strength test. Based on test results, a reduction of 15 °C in mixing temperature was achieved for WMA mixtures compared to HMA using the given chemical additive. The virgin WMA mixture showed superior cracking resistance but lower rutting resistance than HMA, and incorporation of RA material without any further modification in the binder, deteriorated both cracking and rutting performance of WMA. It was also shown that laboratory short-term aging can significantly affect the performance of the mixtures.

## 1. Introduction

The emissions from conventional hot mix asphalt (HMA) production are a complex mixture of fumes, vapors, and solid particulates [1,2]. Although the bitumen used in HMA production contains a much lower concentration of polycyclic aromatic hydrocarbons (PAHs) [3], which are the compounds that have been linked to carcinogenesis [4], the epidemiological studies have found increased lung cancer incidence for occupational exposure to bitumen fumes [5,6]. The studies have shown that levels of PAHs along with other HMA pollutants including, particulate matter (PM), and volatile organic compounds (VOCs), were greatly affected by the temperature of production [7,8]. The efforts made to reduce the temperature of asphalt production will have a direct impact on occupational health. 

Warm mix asphalt (WMA) is one of the ways to lower the production temperature without compromising the workability and performance of the mixture [9,10,11]. The temperature reduction range in WMA is typically around 20–40 °C compared to conventional HMA temperature (140–190 °C) [9]. Though WMA is generally considered to offer benefits like longer hauling distances, reduced binder aging, improved workability, and better working environment for the paving crew [12,13], the type of WMA technology can greatly affect the environmental benefits [14] and the performance [15] of the mixtures.

WMA technologies are classified into three broad categories that include foaming processes, organic additives, and chemical additives [9,11]. Generally, the organic additives have shown a slightly detrimental effect [16,17,18], while chemical additives have improved the moisture resistance of mixtures [18,19,20]. A field performance study found that pavements with three WMA technologies (chemical, foaming, and organic) do not differ significantly in cracking and rutting performance [21]. Multiple studies have confirmed that WMA significantly lowers the emissions and energy consumption compared to HMA production [22,23], but the life cycle studies have shown that the overall environmental impact for WMA can sometimes be equal to that of HMA due to the presence of additives [24,25]. Thus, sustainability evaluation of WMA mixtures becomes important when used alone.

Research shows that the incorporation of RA material greatly reduced the environmental impacts of WMA mixtures [24,26,27]. Additionally, WMA facilitates the incorporation of a high content of reclaimed asphalt (RA) in mixtures and provides synergetic effects on mixture performance [28]. The high RA content mixtures are usually stiffer due to the presence of aged binder, and therefore need changes in the mix design using rejuvenators, additives, or softer binder [29,30,31]. The reduced mixing temperature of WMA limits the undesirable oxidation of bitumen and may compensate for stiff binder present in RA material. In addition, WMA is less affected by aging conditions compared to conventional HMA [32]. Therefore, the use of WMA with RA material can be a viable alternative to conventional HMA, as long-term field performance of WMA mixtures has also been found to be equal to that of HMA mixtures, in terms of cracking and rutting [33].

While some studies show that incorporation of RA significantly improved the moisture damage [15,34,35], others have found that moisture resistance reduced with the incorporation of RA material [36,37,38,39]. This confirms that RA material from various sources may be having a different impact on the moisture performance of the mixture. Generally, the incorporation of RA material has been shown to improve the high-temperature performance of mixture [34,37,40], which is a significant benefit for WMA mixtures, but the RA material incorporation has also been linked to a reduction in low-temperature resistance of the mixtures [35,41].

The extensive research carried out across the globe on warm mix asphalt containing reclaimed asphalt pavement material shows that the performance of mixture can vary with WMA technology type [16,17,18,19,20]; RA content [42]; production temperature [43], and laboratory conditioning [34,44,45]. It becomes important to evaluate the performance of WMA mixtures on a case-to-case basis. The main aim of this paper is to compare the performance of conventional HMA mixture to WMA as well as with WMA containing 60% reclaimed asphalt (RA) content produced using a chemical WMA additive. The comparison of mixtures is based on the evaluation of low-temperature performance, high-temperature performance, and moisture susceptibility characteristics. In addition, the effect of mixture conditioning was also determined by comparing the performance of short-term aged mixtures to unaged mixtures.

## 2. Experimental Plan

**Figure 1:**
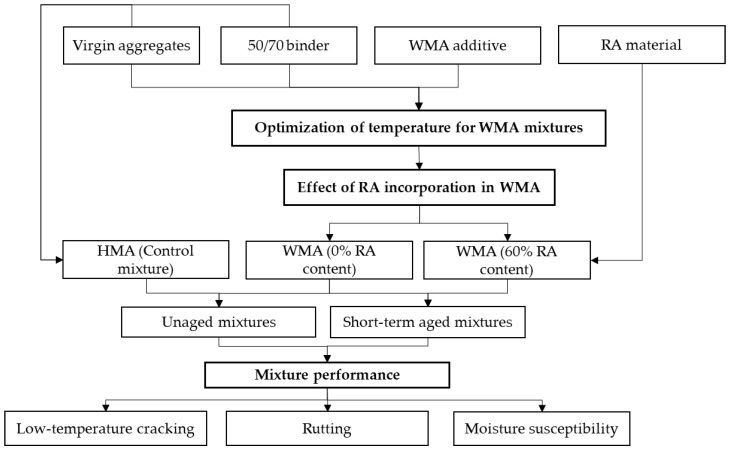
Experimental plan.

The experimental plan developed for this study is in Figure 1. The control mixture for this study was a conventional HMA prepared at a mixing temperature of 140 °C. For WMA, the optimum temperature was determined by preparing mixtures containing chemical additive at a range of mixing temperatures (110, 125 and 140 °C). The optimum temperature was determined based on volumetric requirements for AC-11 surface layer mixtures as given in Latvian road specifications [46]. Finally, the WMA mixtures were produced with 0 and 60% RA content at optimum temperature without any further binder modification. The low-temperature performance, high-temperature performance, and moisture susceptibility were evaluated using tensile stress restrained specimen test, wheel tracking test, and indirect tensile strength test, respectively. All the mixtures were produced using two conditioning methods to compare the unaged mixture to short-term aged mixtures.

## 3. Materials and Methods

### 3.1. Virgin Aggregates and RA Material

**Figure 2:**
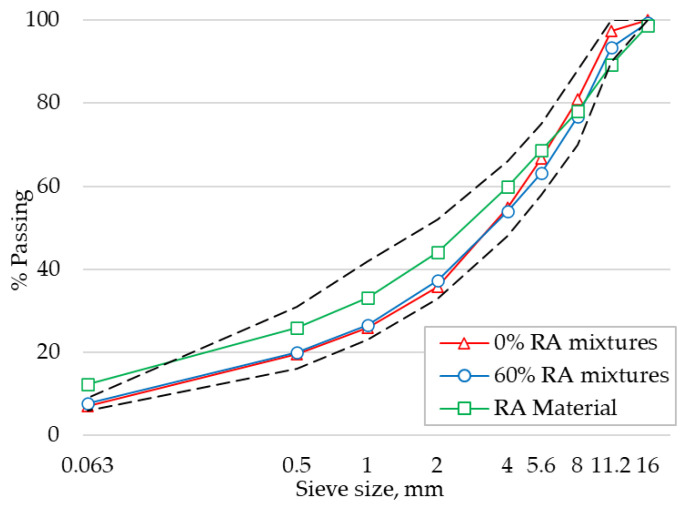
Mixture and extracted RA aggregates gradation.

The virgin aggregates used in the study consisted of Dolomite 8/11, Dolomite 5/8, Dolomite 2/5, Sand 0/4, and filler material. The RA material considered in this study was obtained from an asphalt plant in Vangazi, Latvia, where it was processed using standard operations. The extraction was performed on RA material according to EN 12697-1 [47] standard. The particle size distribution of extracted RA aggregates along with the gradation of mixtures produced in this study is shown in Figure 2. The shape index for coarse aggregates in RA material determined as per EN 933-4 [48] was found to be 12. The flow coefficient for sand and fine RA aggregates determined according to EN 933-6 [49] were 31.5 and 28, respectively.

### 3.2. Virgin Binder and RA Binder

The virgin binder used in this study was a 50/70 penetration grade bitumen obtained from ORLEN Asfalt, Mazeikiai, Lithuania. The RA binder was recovered from bitumen solution using a rotary evaporator according to EN 12697-3 [50] standard. The RA material had a binder content of 4.35% and the penetration of RA bitumen was 39 × 0.1 mm. In this study, a chemical additive widely used in industry was utilized which is based on its surfactant properties. Typically, with this additive, the production temperature range is 85–115 °C for a dosage of 0.5% w/b [10]. In this study, the supplier recommended dosage of 0.4% w/b was used for all the WMA mixtures at different temperatures. The control HMA mixture used in this study was designed using the Marshall mix design method and the optimum binder content was determined as 5.5% by weight of aggregates. The binder content for virgin WMA and WMA containing 60% RA was 5.5% by weight of aggregates. For WMA mixtures containing 60% RA material, the aggregate gradation similar to virgin mixtures was achieved by proportioning the virgin aggregates. For these mixtures, the binder quantity was calculated by deducting the amount of reclaimed asphalt binder from the total binder requirement of WMA mixtures.

### 3.3. Mixture Preparation

The virgin aggregates and RA materials for all the mixtures were heated at their respective mixing temperature for 2.5 h in the oven. The WMA binder was prepared manually using a paddle mixer. For this, the 50/70 binder was heated at a temperature of 150 °C in the oven for one hour, and the required dosage of additive was added to the binder followed by mixing for 5 min. Prior to asphalt mixing, virgin binder and WMA binder were heated for 2.5 h in the oven at respective mixing temperatures of the mixture. Initially, all the constituent virgin aggregates, and RA material were mixed without bitumen in the mixer for 1 min to ensure a homogeneous blend of aggregates as well as to increase the activation of the RA binder. After 1 min, the bitumen was added, and mixing was continued for another 4 min. The unaged mixtures were compacted immediately after the mixing. The compaction temperature was 5 °C lower than the mixing temperature for all the mixtures. For simulating short-term aging, mixtures were conditioned in an oven with a covered pan for 4 h according to EN 12697-52 [51] standard. HMA mixtures were conditioned at a temperature of 135 °C and WMA mixtures were conditioned at 120 °C. The conditioning temperature for short-term aging was kept 5 °C lower than the mixing temperature to consider the loss of temperature during the production stage. All the mixtures produced in this study are shown in Table 1. It should be noted that “WMA” only refers to the mixtures that are produced using the chemical additive, and the temperature for each mixture is indicated in Table 1.

### 3.4. Thermal Stress Restrained Specimen Test (TSRST)

Thermal stress restrained specimen test (TSRST) was performed according to EN 12697-46 [52] to determine the low-temperature cracking performance of the mixtures. The test is conducted by keeping the length of the specimen constant and reducing the chamber temperature until the sample generates cracks due to thermal stress. The initial test temperature is 20 °C and the temperature reduction rate is 10 °C/h until failure occurs. The specimens for the test were prepared by sawing the slabs to the required dimensions (160 mm × 50 mm × 50 mm). For each mixture, two specimens were tested, and the average value was reported.

### 3.5. Wheel Tracking Rest

The wheel tracking test was conducted in dry condition according to EN 12679-22 [53] to determine the rutting susceptibility of the mixtures. This test was conducted at 60 °C by applying a load of 700 N using a rubber tire and recording the rut depth using two linear variable deformation transducers (LVDT). The test was run up to 10,000 load cycles. The results of this test indicate rut depth for a single sample tested for each mixture. In addition, wheel tracking slope was also calculated from Equation (1) using rut depth obtained at 5000 and 10,000 load cycles.

(1) $$WTS=\frac{\left(d_{10,000}-d_{5000}\right)}{5}$$

where,

*WTS* is the wheel-tracking slope per 10^3^ load cycles in mm, *d*_5,000_ and *d*_10,000_ are the rut depths in mm after 5000 and 10,000 load cycles, respectively.

### 3.6. Moisture Susceptibility Test

The indirect tensile strength test was carried out to determine the effect of reduced temperature on moisture susceptibility of mixtures according to EN 12697-12 [54]. For moisture susceptibility evaluation, Marshall samples were prepared using 35 blows on each side. According to the standard, a test temperature of 5–25 °C can be used for moisture susceptibility evaluation. In this study, a test temperature of 22 °C was used which was also the measured room temperature, and this was selected to avoid the temperature changes in the specimen during the test. The indirect tensile strength of mixtures was evaluated according to EN 12697-23 [55]. The average of three specimens is reported in the results. The ratio of the indirect tensile strength of wet specimens and dry specimens is calculated and expressed as a percentage to determine the moisture damage in the mixtures.

## 4. Results and Discussion

### 4.1. Volumetric Analysis

The volumetric analysis test results for all the mixtures are given in Table 2. WMA0-140-UN mixture showed lower air voids as compared to the HMA0-140-UN mixture. In this case, the incorporation of additive without changing the temperature enhanced the compactibility of the mixture. This was an expected observation as, the chemical additives are well known to improve the coating, workability, and compactibility of the mixtures [9,10,11]. This shows that the chemical additives can be incorporated in the HMA mixtures without lowering the temperature, when the main aim is to enhance the compactibility of the mixture.

As observed in Table 2, WMA0-125-UN showed higher air voids than WMA0-140-UN, and the WMA0-110-UN mixture showed higher air voids than the WMA0-125-UN mixture. The reduction in mixing temperature of WMA mixtures showed an increase in air voids in the mixture. This may be due to the fact that with the reduction in temperature, the viscosity of bitumen is increased. As a result of increased viscosity, the compactibility is reduced. The Latvian Road specifications [46] require the air voids for AC-11 surface layer mixtures to be in the range of 1.5–4%. As seen in Table 2, only the WMA0-125-UN mixture meets the requirement for the air voids among all WMA mixtures without RA material. Based on these results, the optimum mixing temperature for WMA mixtures was selected as 125 °C. The subsequent WMA mixtures for performance evaluation were produced at a temperature of 125 °C in this study.

**Figure 3:**
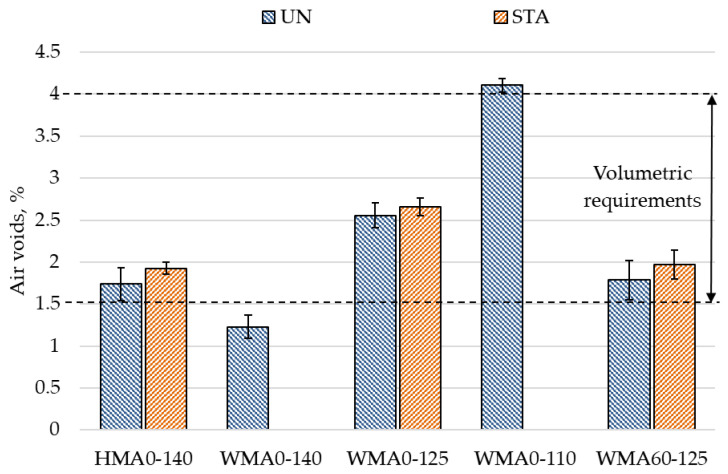
Volumetric analysis results.

As seen in Figure 3, the air voids for the unaged mixtures were not significantly different from short-term aged mixtures. This indicates that even though the binder gets oxidized during 4 h short-term aging process and may become stiffer, the change is not enough to significantly affect the compaction characteristics of mixtures.

The WMA60-125-UN mixture showed 0.8% lower air voids as compared to the WMA0-125-UN mixture, and the WMA60-125-STA mixture showed 0.6% lower air voids as compared to the WMA0-125-STA mixture. This shows that the incorporation of 60% RA material into WMA resulted in reduced air voids in the mixture. The lower air void content is a typical problem for high content RA mixtures [56]. Considering, that the gradation of RA mixtures is the same as virgin mixtures in this study, it is likely that lower air voids in RA mixtures may be due to the presence of unbound fines from RA that did not mix well with the binder in the mixture. These unbound fines are generated during the milling process due to the crushing of aggregates.

### 4.2. Low-Temperature Performance

**Figure 4:**
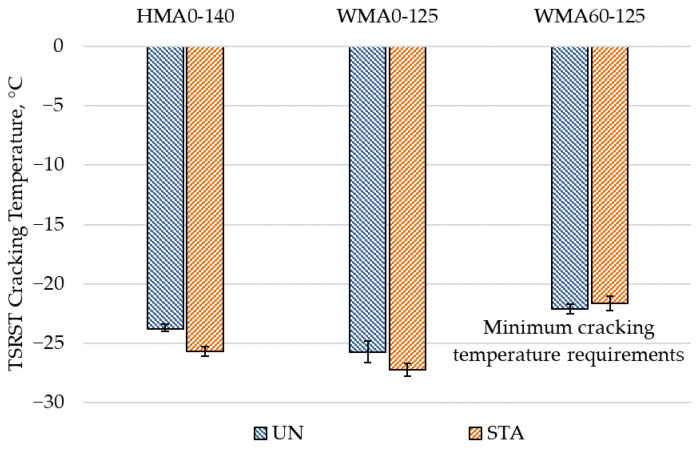
Low-temperature cracking test results.

For mixtures produced in colder regions, low-temperature cracking is an important concern. Moreover, the inclusion of RA material increases the low temperature cracking susceptibility of mixtures due to presence of aged RA binder. The results of low temperature cracking test are shown in Figure 4. A lower fracture temperature indicates better performance in low-temperature cracking. It can be observed that WMA mixtures (WMA0-125-UN and WMA0-125-STA) showed lower cracking temperature (1.9 and 1.55 °C lower) than HMA mixtures (HMA0-125-UN and HMA0-125-STA) mixtures. This indicates that WMA mixtures are more resistant to low-temperature cracking compared to HMA mixtures. The modified binder in WMA mixtures is subjected to lower mixing temperature compared to the HMA binder which results in lower binder aging in WMA. The lower binder aging in WMA may be the reason for the better low-temperature performance of these mixtures.

It was expected that the short-term aging of the mixtures will degrade the low temperature cracking resistance of the mixtures as a result of oxidation of binder. However, the short-term aged mixtures (HMA0-140-STA and WMA0-125-STA) showed slightly lower cracking temperature (1.85 and 1.5 °C lower) compared to unaged mixtures (HMA0-140-UN and WMA0-120-UN). It is believed that this change might be due to the variability of materials, as the fracture temperature for TSRST is known to be highly sensitive to aggregate and asphalt type [48]. This variability could also have come from glue due to stress concentration at the epoxy region in the specimen. Nevertheless, for these mixtures, WMA showed superior cracking resistance than HMA, which follows the same trend as unaged mixtures.

It can be seen that the incorporation of 60% RA material has substantially degraded the low-temperature cracking resistance of WMA mixtures. WMA60-125-UN and WMA60-125-STA mixtures showed significantly lower cracking temperatures (3.6 and 5.9 °C, respectively) compared to WMA0-125-UN and WMA0-125-STA mixtures. This is due to the presence of stiff oxidized binder from RA material that is ultimately making the mixture more brittle. The fine aggregates in RA are also more rounded than the virgin aggregates (flow coefficient value for RA mixtures was 28 compared to 31.5 for virgin mixtures), which could be another reason for the lower fracture resistance of these mixtures. As a result, the mixtures containing RA material could not fulfill the minimum requirement of −22.5 °C for low temperature cracking for AC-11 surface layer mixtures as per Latvian Roads specifications [46]. Since the aim of this study was to isolate the effect of WMA technology, no further modifications in the binder were performed for the high RA mixture. However, it is clear that the additive alone is not sufficient, and the addition of a rejuvenator or softer binder may be required to improve the low-temperature cracking performance of high content RA mixtures.

The low temperature cracking resistance deteriorated on RA material incorporation which is in agreement with past studies [35,41]. Considering that 20–30% RA incorporation has not shown a reduction in low-temperature cracking [20,57,58], lower RA content may be used with WMA to improve the low-temperature performance. However, the long-term performance of RA mixtures in low-temperature cracking needs to be evaluated.

### 4.3. Rutting Performance

**Figure 5:**
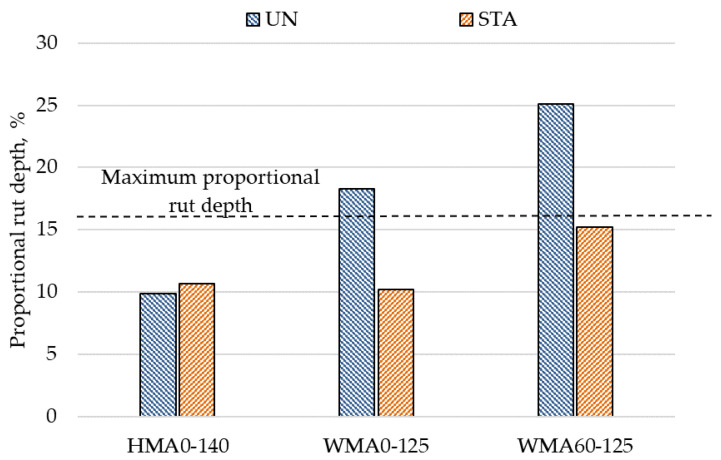
Proportional rut depth results.

Figure 5 shows the results of the wheel tracking test. It can be observed that proportional rutting depth for WMA0-125-UN mixture was 8.4% higher compared to rut depth in HMA0-140-UN mixture. The binder in WMA mixtures is softer than the virgin binder due to presence of the additive. The reduced stiffness of mixture due to binder modification increased the rutting susceptibility in WMA compared to HMA. It was also observed in past studies that WMA mixtures were more susceptible to rutting failure due to reduced aging of bitumen in the mixture [12,59,60].

The unaged WMA mixture containing 60% RA material showed the highest rutting potential among all the mixtures. The incorporation of 60% RA material increased the rutting depth by 6.8% in the WMA mixture. This is opposite to the general trend observed from past studies, where the rutting resistance was increased with the incorporation of RA material [34,37,40]. One reason for the lower rutting resistance of RA mixture could be the low angularity of fine aggregates in RA material as the flow coefficient for fine RA aggregates was lower compared to the fine aggregates in the virgin mixture. Another reason may be lower degree of blending between RA binder and virgin binder ultimately resulting in an unstable mixture.

As shown in Figure 5, the short-term aging did not show any effect on the rutting performance of control HMA mixture. However, for the virgin WMA mixture, the rutting depth was considerably reduced on short-term aging to a level equal to that of control HMA mixture. For the 60% RA mixture, the rut depth was still 5% higher than the virgin WMA mixture in aged condition. The short-term aging of the WMA mixture may have resulted in oxidation of the binder which made the mixture stiffer than the unaged mixture. A past study has also shown that aged mixtures show higher stiffness and better rutting resistance than unaged mixtures [45]. Both the unaged WMA mixtures (WMA0-125-UN and WMA60-125-UN) could not fulfill the proportional rut depth requirement. However, the short-term aged WMA mixtures fulfilled the proportional depth rutting criteria.

**Figure 6:**
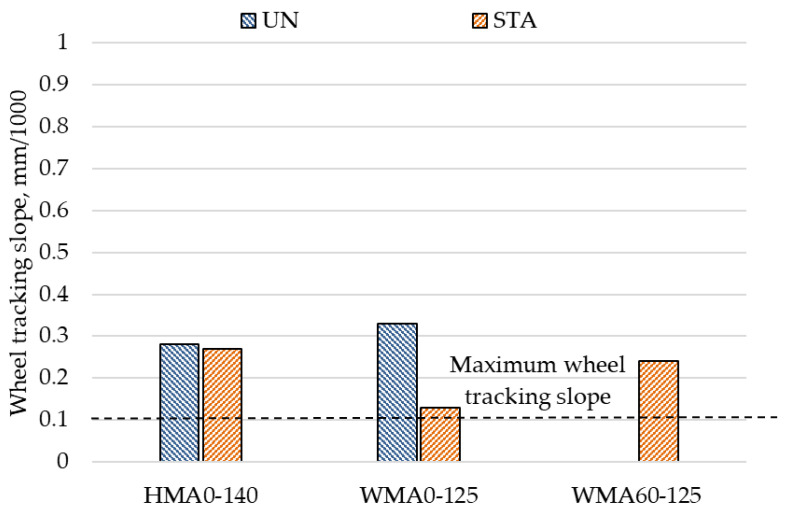
Wheel tracking slope results.

The Latvian Road specifications [46] have set the requirement of a minimum of 0.1 mm/1000 for wheel tracking slope for the highest traffic level. None of the mixtures produced in the study fulfilled the wheel tracking slope criteria as shown in Figure 6. The short-term aging of the WMA mixture without the RA material has significantly reduced the wheel tracking slope which shows the importance of taking into account the effect of the conditioning process while evaluating the rutting performance of the mixtures.

### 4.4. Moisture Susceptibility 

**Figure 7:**
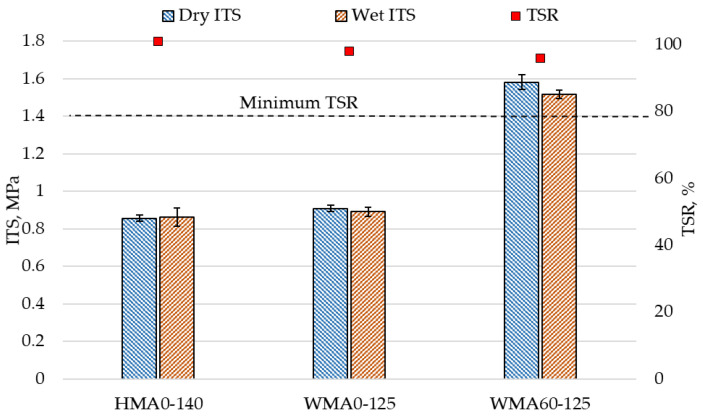
Moisture susceptibility results.

The results of the indirect tensile strength test for all the mixtures are shown in Figure 7. It can be seen that indirect tensile strength for all the mixtures is not significantly changed after the moisture conditioning. This shows that reduction of temperature in WMA did not have any adverse effect on moisture performance. As a result, all the mixtures fulfilled the minimum tensile strength ratio (TSR) criteria of 80%. The WMA mixture containing 60% RA material (WMA60-125) showed 74% higher dry ITS and 70% higher wet ITS compared to the virgin WMA mixtures. This is due to the presence of the oxidized binder from RA material that made the mixture stiffer and increased the indirect tensile strength of the mixture. The incorporation of RA material did not show a significant change in moisture susceptibility of WMA mixtures.

## 5. Conclusions

This study compared the laboratory performance of HMA mixture with virgin WMA mixture and WMA mixtures 60% RA material. The performance was evaluated using tensile stress restrained specimen test, wheel tracking test, and indirect tensile strength test. For all the mixtures, the performance was evaluated for unaged and short-term aged mixtures. Based on this study, the following conclusions are made:

- The addition of chemical additive without changing the temperature enhanced the compactibility of the HMA mixture. A reduction of 15 °C in mixing temperature compared to HMA mixture was achieved for WMA mixtures using chemical additive. The incorporation of RA material reduced the air voids in the WMA mixture.
- The low temperature cracking performance of virgin WMA mixtures was superior to control HMA mixture. However, the addition of RA material degraded the low-temperature cracking resistance of the WMA mixture.
- The virgin WMA mixture showed higher rutting potential compared to the control mixture, due to binder modification and lower binder aging. The incorporation of RA material degraded the rutting resistance of the WMA mixture. The short-term aging of WMA mixtures also affected the rutting resistance compared to unaged mixtures.
- The reduction in mixing temperature using chemical additive and incorporation of RA material did not show any negative effect on moisture characteristics of the mixtures. The indirect tensile strength for WMA mixture containing RA material was much higher than HMA and virgin WMA mixtures, due to the presence of oxidized RA binder.

More research is required to study the effect of the combination of rejuvenators and WMA additives on the activation of RA binder. However, careful selection of rejuvenator is required due to rutting concerns of mixtures, as observed in this study. Additionally, the conventional methods for designing mixtures may not be completely applicable to high RA mixtures due to high variability in properties of RA materials originating from different sources. Therefore, future studies should also look into developing performance-based specifications for the design of high RA content mixtures.

## Figures and Tables

**Table 1:** Mixture information.

Mixture	RA Content	Mixing Temperature	Conditioning Process
HMA0-140-UN	0%	140 °C	Unaged
WMA0-140-UN	0%	140 °C	Unaged
WMA0-125-UN	0%	125 °C	Unaged
WMA0-110-UN	0%	110 °C	Unaged
HMA0-140-STA	0%	140 °C	Short-term aged @ 135 °C for 4 h
WMA0-125-STA	0%	125 °C	Short-term aged @ 120 °C for 4 h
WMA60-125-UN	60%	125 °C	Short-term aged @ 120 °C for 4 h
WMA60-125-STA	60%	125 °C	Short-term aged @ 120 °C for 4 h

**Table 2:** Volumetric analysis results.

Mixture	Max Density, kg/m^3^	Air Voids, %	VMA, %	VFB, %
HMA0-140-UN	2487	1.7	15	88.4
WMA0-140-UN	2493	1.2	14.7	91.2
WMA0-125-UN	2501	2.6	15.8	84.1
WMA0-110-UN	2523	4.1	17.2	76.4
HMA0-140-STA	2518	1.9	15.4	87.7
WMA0-125-STA	2496	2.7	15.2	87.5
WMA60-125-UN	2494	1.8	15.9	83
WMA60-125-STA	2507	2	15.5	86.5

## Data Availability

Data sharing is not applicable.
